# Effect Size of Targeted Temperature Management in Pediatric Patients with Post-Cardiac Arrest Syndrome According to the Severity

**DOI:** 10.3390/life15010026

**Published:** 2024-12-30

**Authors:** Takeshi Namba, Mitsuaki Nishikimi, Ryo Emoto, Kazuya Kikutani, Shinichiro Ohshimo, Shigeyuki Matsui, Nobuaki Shime

**Affiliations:** 1 Department of Emergency and Critical Care Medicine, Graduate School of Biomedical and Health Sciences, Hiroshima University, Hiroshima 739-0046, Japan; namba1t@hiroshima-u.ac.jp (T.N.); kikutani@hiroshima-u.ac.jp (K.K.); ohshimos@hiroshima-u.ac.jp (S.O.); shime@koto.kpu-m.ac.jp (N.S.); 2 Department of Biostatistics, Graduate School of Medicine, Nagoya University, Nagoya 464-8603, Japan; remoto@med.nagoya-u.ac.jp (R.E.); smatsui@med.nagoya-u.ac.jp (S.M.)

**Keywords:** children, national registry, neurological prognosis, out-of-hospital cardiac arrest (OHCA), revised post-cardiac arrest syndrome for therapeutic hypothermia score (rCAST)

## Abstract

Aim: Few studies have investigated the differential effects of targeted temperature management (TTM) according to the severity of the condition in pediatric patients with post-cardiac arrest syndrome (PCAS). This study was aimed at evaluating the differential effects of TTM in pediatric patients with PCAS according to a risk classification tool developed by us, the rCAST. Methods: We used data from a nationwide prospective registry for out-of-hospital cardiac arrest (OHCA) patients in Japan. We classified eligible pediatric PCAS patients (aged ≤ 18 years) into quintiles based on their rCAST scores and evaluated the effect of TTM on the neurological outcomes in each severity group. Then, focusing on the severity group that appeared to benefit from TTM, we also evaluated the effect of TTM by propensity score analysis. Good neurological outcome was defined as a score on the Cerebral Performance Category or Pediatric Cerebral Performance Category scale of ≤2 at 30 days. Results: Among 1526 OHCA pediatric patients enrolled in the registry, the data of 307 PCAS patients were analyzed. None of the patients in the fifth quintile (rCAST ≥ 18.5) showed a good neurological outcome, regardless of whether they received TTM or not (0% [0/20] vs. 0% [0/73]). The propensity score analysis showed that TTM was significantly associated with a good neurological outcome in patients with rCAST scores in the first to fourth quintile (odds ratio: 1.21 [1.04–1.40], *p* = 0.014). Conclusions: TTM was significantly associated with good neurological outcomes in pediatric PCAS patients with rCAST scores of ≤18.0.

## 1. Introduction

Pediatric out-of-hospital cardiac arrest (OHCA) is a rare event and is associated with a poor neurological outcome [1]. In clinical practice, therapeutic hypothermia has been employed frequently to reduce the neurological damage after cardiac arrest (CA) through decreasing the cerebral metabolic rate and inhibiting the molecular pathways that can exacerbate neuronal damage [2]. Although evidence of the beneficial effects of therapeutic hypothermia is based on previous studies conducted in adults [3], children [4,5,6,7], and neonates [8], recent randomized clinical trials (RCTs) have failed to show any superiority of therapeutic hypothermia over maintaining normothermia for pediatric CA patients [9,10]. Today, the most recent statement from the International Liaison Committee on Resuscitation for the post-resuscitation care of pediatric OHCA patients recommends targeted temperature management (TTM), including therapeutic hypothermia and maintenance of normothermia [9,10].

Although TTM has the potential to improve a good neurological outcome in pediatric OHCA patients, the medical costs of TTM are a serious concern. According to a previous study, TTM increases the hospital care costs by $5444–8035 USD [11]. Considering the invasiveness of TTM [12], as well as the fact that only 0.3% to 4% of pediatric OHCA patients show favorable neurological outcomes [1], it may be important to identify particular subgroups of patients based on the severity score that would benefit from TTM.

Previously, we developed a revised version of the “post-Cardiac Arrest Syndrome for Therapeutic hypothermia scoring system” (rCAST) for risk classification for post-cardiac arrest syndrome (PCAS) patients [13]. The rCAST was reported to be useful for predicting the neurological outcome of PCAS patients in several countries [14,15,16,17]. Recently, the rCAST was also validated for pediatric OHCA patients [18], but it was unknown whether the rCAST score could be also useful for identifying subgroups of pediatric PCAS patients that could derive benefit from TTM. The aim of this study was to evaluate the differential effects of TTM in pediatric PCAS patients according to the score points on the rCAST.

## 2. Materials and Methods

### 2.1. Design

In this cohort study, we conducted a retrospective analysis of the data of patients registered in a nationwide prospective registry of OHCA patients across Japan (a total of 157 institutions), the Japanese Association of Acute Medicine (JAAM-OHCA registry). The profile of the JAAM-OHCA registry data has been reported previously [19]. Briefly, emergency medical service personnel collect pre-hospital data based on the Utstein-style template [20,21], and physicians at the participating institutions collect in-hospital data, including the data of their outcomes. This study was conducted with the approval of the Institutional Review Boards of all the participant institutions; the Institutional Review Boards of all institutions waived the requirement for obtaining informed patient consent from the study participants to ensure participant anonymity, as stipulated in the Japanese government guidelines.

### 2.2. Patients

The data of pediatric OHCA patients (≤18 years old) who achieved the return of spontaneous circulation (ROSC) between 1 June 2014, and 31 December 2020 were included in this study. We divided all analyzed patients into those who underwent TTM (TTM group) and those who did not (no-TTM group). Patients with any missing values of the variables needed for the calculation of the rCAST scores (described below) or for adjustments in the analyses were excluded (complete case analyses). Basically, we performed TTM according to the recommendation of Japanese resuscitation guidelines [22]. TTM was performed for pediatric CA patients who were in comas, which is defined as giving no meaningful responses to any questions, after achieving ROSC. Cooling devices included gastric cooling, cold intravenous fluid, surface cooling with a feedback system, intravascular cooling with a feedback system, and so on. The setting temperatures during TTM were between 32–36 °C, typically for 24 h. Ketamine, dexmedetomidine, midazolam, fentanyl, and rocuronium were used for sedation, analgesia, and muscle relaxation, respectively, according to the individual attending clinicians’ preferences.

### 2.3. Severity Scale for Pediatric PCAS Patients

The rCAST score was utilized as a risk stratification tool in this study. The calculation method was described in a previous report [23]. The rCAST score is calculated prior to the initiation of TTM based on five clinical parameters (initial rhythm, witnessed cardiac arrest, time until ROSC, blood pH and serum lactate level at hospital arrival, and the motor scale of GCS at the time of ROSC) [23]. The rCAST has demonstrated an excellent predictive accuracy for neurological outcomes of pediatric patients with OHCA [18]. We classified eligible pediatric PCAS patients for this study into quintiles based on the rCAST scores to evaluate the beneficial effects of TTM according to the severity of PCAS.

### 2.4. Endpoints

The primary outcome was the percentage of patients with a good neurologic outcome at 30 days. We classified the neurologic outcomes according to the scores on the Cerebral Performance Category scale (CPC) for patients over 18 years old (CPC 1, full recovery; CPC 2, moderate disability; CPC 3, severe disability; CPC 4, coma or vegetative state; CPC 5, died) or on the Pediatric Cerebral Performance Category scale (PCPC) for patients under 18 years old (PCPC 1, full recovery; PCPC 2, mild disability; PCPC 3, moderate disability; PCPC 4, severe disability; PCPC 5, coma or vegetative state; and PCPC 6, died) at 30 days. CPC1/PCPC1 and CPC2/PCPC2 are considered as representing good neurologic outcomes [24,25]. We also evaluated the survival rate at 30 days as a secondary outcome.

### 2.5. Statistical Analysis

We performed the Chi-squared test and Student’s *t*-test to compare the categorical and continuous variables, respectively. The predictive accuracy of the rCAST score was evaluated in all the analyzed patients by using the area under the receiver operating characteristic curve (AUC). The calibration was also assessed by plotting the agreement between the observed outcomes and predicted probabilities, as well as Hosmer and Lemeshow’s goodness of fit test. Not only did we perform the comparison analyses based on the quintile on the rCAST score, but we also plotted adjusted spline curves showing how the effects of TTM were modified by the score points on the rCAST score. All adjustment variables were previously reported as potentially exerting a significant influence on their neurological outcomes. The variables are as follows: ((1) age, (2) sex [26], (3) initial rhythm [27], (4) time until ROSC [28,29], (5) the motor scale of GCS ≥ 2 [30], and (6) ECPR cases or not [31]). In the inverse-probability treatment weighting (IPTW) analysis, we used the same six adjustment variables to determine the propensity score. We used R (version 4.1.1) package mgcv for plotting spline curves (R Foundation for Statistical Computing, Vienna, Austria). All other analyses were conducted using the JMP pro, version 17.0.0. (SAS Institute Inc., Cary, NC, USA). All reported *p* values were two-sided, and the statistical significance was set at *p* < 0.5.

## 3. Results

**Figure 1:**
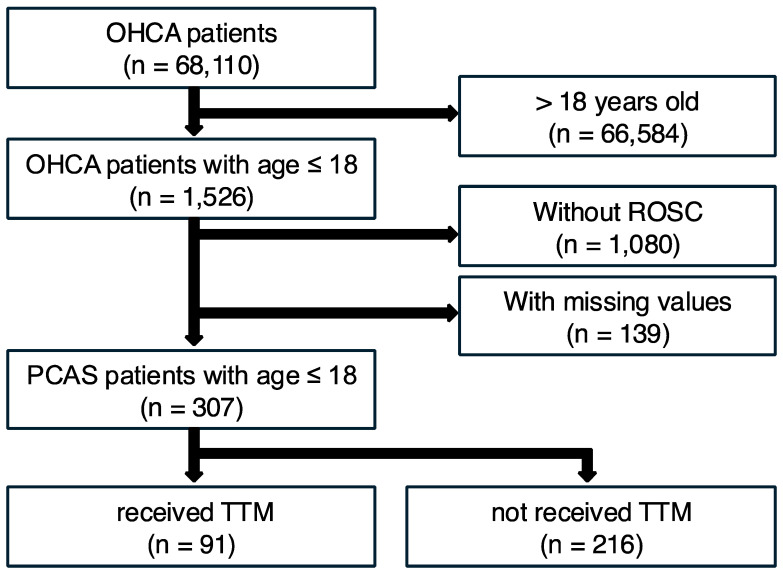
Patient flow in this study. Of a total of 68,110 OHCA patients, 307 were selected for this analysis. Of these, 91 patients (29.6%) received TTM, and 216 patients (70.4%) did not receive TTM. OHCA = out-of-hospital cardiac arrest; ROSC = return of spontaneous circulation; PCAS = post-cardiac arrest syndrome; TTM = target temperature management.

Among the 68,110 OHCA patients in the JAAM-OHCA registry between 1 June 2014, and 31 December 2020, 1526 were pediatric patients (≤18 years old). Of these, 1219 were excluded from this study because ROSC was not achieved (n = 1080) or they had missing values for the analysis in this study (n = 139). Finally, the data of the remaining 307 pediatric PCAS patients divided into the TTM group (n = 91) and the no-TTM group (n = 216) were analyzed (Figure 1).

The baseline characteristics of the eligible patients included in this study are summarized in Table 1. While no statistically significant differences in variables such as age and sex were observed between the TTM group and no-TTM group, there were significant differences in variables such as the primary cause of cardiac arrest, initial rhythm, use of ECMO, and time until ROSC between the two groups. The median rCAST score was 16.0 (11.5–18.0) in the TTM group and 16.5 (15.0–18.5) in the no-TTM group, the difference between the two groups not being statistically significant. Twenty-six of the 91 patients of the TTM group (28.6%) and 23 of the 216 patients in the no-TTM group (10.7%) showed good neurological outcomes at 30 days.

At first, we confirmed that the rCAST score could be useful as a risk classification tool for our analyzed patients. The AUC of rCAST for their poor neurological outcomes at 30 days was 0.91 (0.85–0.97) (Supplementary Figure S1). The calibration plots demonstrated good agreement between the observed and predicted probabilities (Hosmer and Lemeshow’s goodness of fit test; *p* = 0.71) (Supplementary Figure S2).

**Figure 2:**
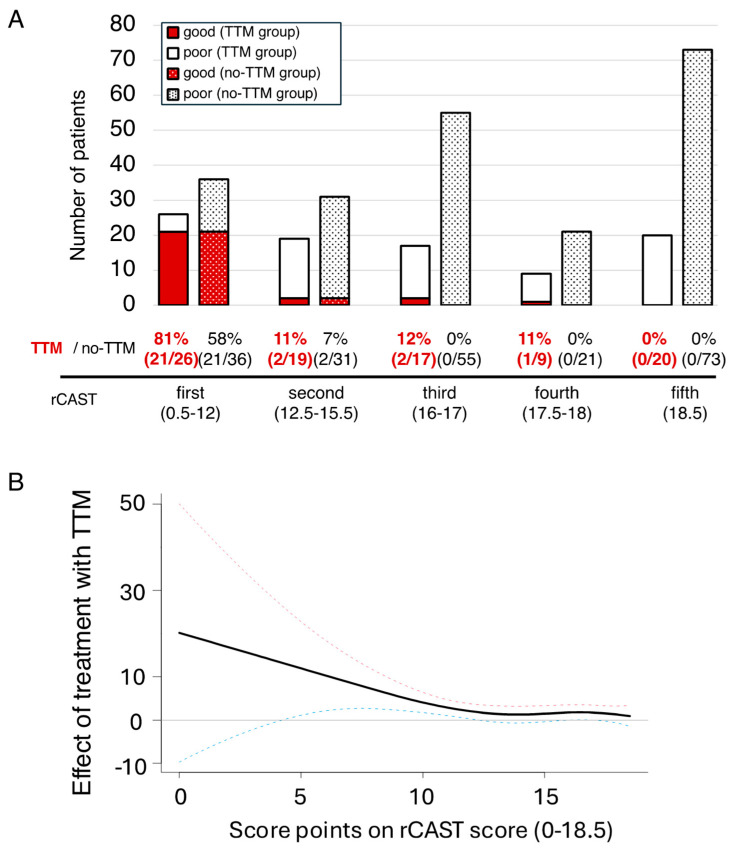
Good neurological outcome at 30 days according to the rCAST score: (**A**) The percentages of patients with good neurological outcomes at 30 days in the TTM group and no-TTM group according to the PCAS severity (rCAST quintile). (**B**) Adjusted spline curve depicting the relationship between the effects, in terms of the logarithm of the odds ratio, of TTM versus no-TTM and the rCAST score. We evaluated the estimated effect size of a good neurological outcome at 30 days. The black line is the spline curve, whereas the red and blue dotted lines are the 95% upper limit and lower limit lines, respectively. TTM = target temperature management; PCAS = post-cardiac arrest syndrome; rCAST = revised post-cardiac arrest syndrome for therapeutic hypothermia.

**Figure 3:**
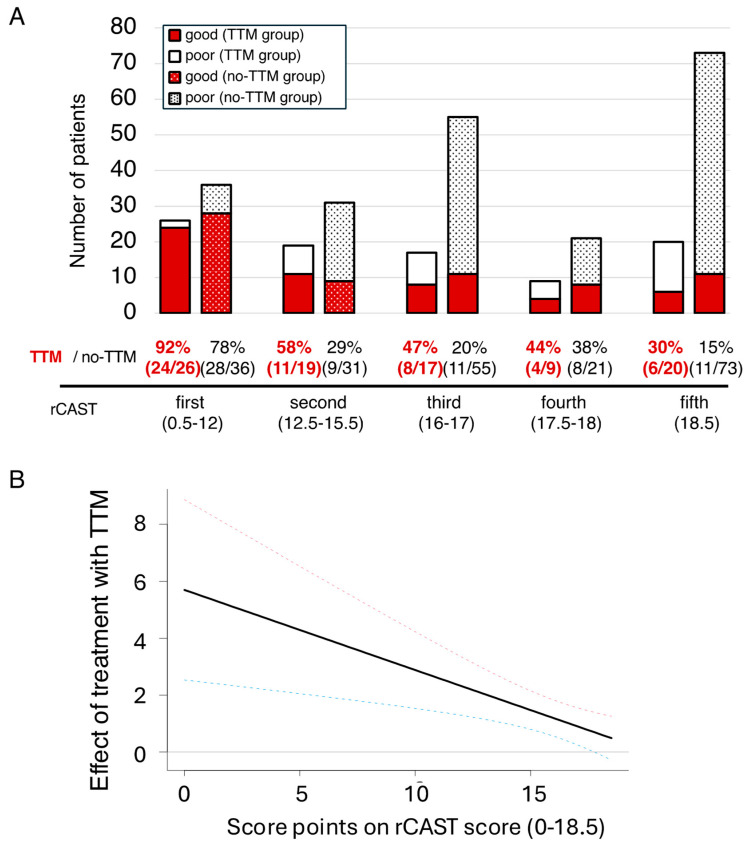
Survival outcome at 30 days according to the rCAST score: (**A**) The percentages of patients with survival at 30 days in the TTM group and no-TTM group according to the PCAS severity (rCAST quintile). (**B**) Adjusted spline curve showing the relationship between the effect, in terms of the logarithm of the odds ratio, of TTM versus no-TTM and the rCAST score. We evaluated the estimated effect size of the survival outcome at 30 days. TTM = target temperature management; PCAS = post-cardiac arrest syndrome; rCAST = revised post-cardiac arrest syndrome for therapeutic hypothermia.

The 91 patients in the TTM group and 216 patients in the no-TTM group were then divided into quintiles based on the rCAST scores as follows: first quintile: rCAST score 0.5–12; n = 26, and n = 36, respectively; second quintile: rCAST score 12.5–15.5; n = 19 and n = 31, respectively; third quintile: rCAST score 16–17; n = 17 and n = 55, respectively; fourth quintile: rCAST score 17.5–18; n = 9 and n = 21, respectively; fifth quintile, rCAST score 18.5; n = 20 and n = 73, respectively. As for the outcomes in each of the quintiles of the TTM group vs. the no-TTM group, the rate of patients with good neurologic outcome at 30 days was 81% (21/26) vs. 58% (21/36) in the first quintile, 11% (2/19) vs. 7% (2/31) in the second quintile, 12% (2/17) vs. 0% (0/55) in the third quintile, 11% (1/9) vs. 0% (0/21) in the fourth quintile, and 0% (0/20) vs. 0% (0/73) in the fifth quintile (Figure 2A). The baseline characteristics of the patients in each quintile are summarized in Supplementary Tables S1 and S2. We also plotted adjusted spline curves as another approach for evaluating how the effect size of TTM was different depending on the rCAST score (Figure 2B). The results showed that TTM was associated with higher rates of good neurologic outcomes at 30 days in the patients with lower score points on the rCAST. The analyses for survival at 30 days, which had similar trends as those for the neurological outcomes, were shown in Figure 3.

**Figure 4:**
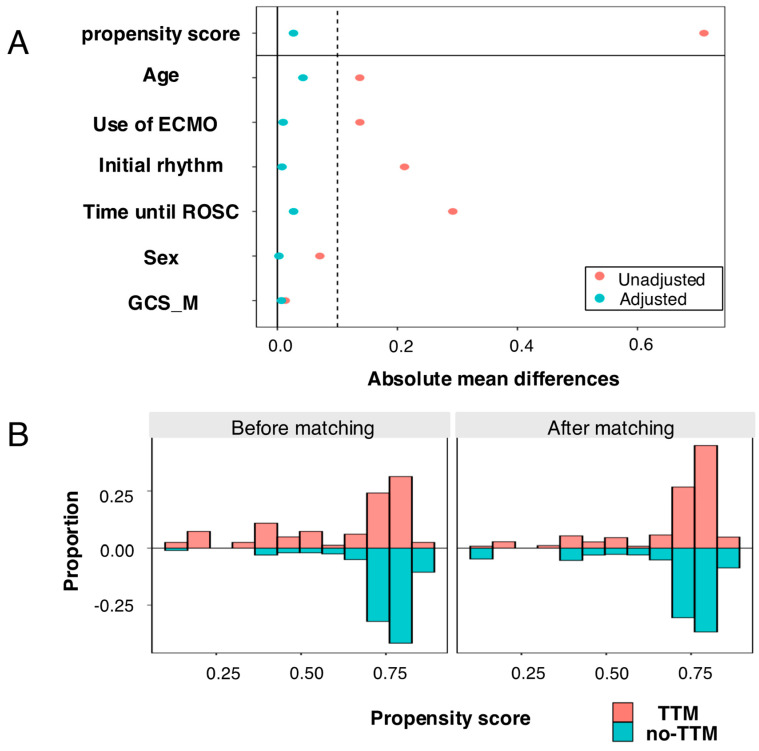
Covariate balance and distributional balance for propensity score: (**A**) Absolute mean differences in the TTM group versus the non-TTM group before and after the inverse probability of treatment weighting adjustment. (**B**) Distribution of propensity scores before and after matching for age, use of ECMO, initial rhythm, time until ROSC, sex, and the motor scale of GCS. ECMO = extracorporeal membrane oxygenation; ROSC = return of spontaneous circulation; GCS_M = motor scale of the Glasgow coma scale; TTM = target temperature management.

Because a trend indicating that TTM may be beneficial for all but the fifth quintile pediatric PCAS patients was observed, as shown in Figure 2, we conducted an IPTW analysis to evaluate the association between the neurological outcome at 30 days and TTM, focusing on patients in the first to fourth quintiles. The covariate balance and distributional balance for the propensity score are shown in Figure 4. The results showed that TTM was significantly associated with good neurological outcomes (OR; 1.21 [1.04–1.40], *p* = 0.014) in the first to fourth quintiles.

## 4. Discussion

In adults, several studies have shown that the effectiveness of TTM at lower target temperatures varies depending on the severity of PCAS [32,33,34]. While these findings cannot be directly applied to children, they support our hypothesis that the effects of TTM in children may also vary based on the severity of PCAS. In this observational study, we successfully demonstrated that TTM was significantly associated with good neurological outcomes in pediatric PCAS patients with rCAST scores of ≤18.0, while none of the patients with rCAST scores of ≥18.5 showed good neurological outcomes regardless of whether they received TTM or not. Considering the medical costs and invasiveness of TTM, we propose that the patients with rCAST scores of ≥18.5 may not be candidates for TTM.

We identified the beneficial effects of TTM in pediatric PCAS patients with rCAST scores of ≤18.0. Many animal studies have reported that TTM, including therapeutic hypothermia, may provide no benefit if the brain ischemia has progressed irreversibly and there is no room for potential recovery by preventing fever [35,36]. The rCAST scores of ≤18.0, on the other hand, may imply that there is some room for recovery and that TTM may have the potential to improve neurological outcomes in these patients, although further prospective studies are needed.

A previous study of adult OHCA patients showed that therapeutic hypothermia as compared with normothermia treatment was significantly associated with good neurological outcomes of PCAS patients with moderate severity but not in the low- or high-severity groups [34]. Although our present study was conducted to compare the effects of TTM versus no-TTM, and not therapeutic hypothermia versus normothermia treatment, there may be a discrepancy in the differential responses to treatment according to the severity of the brain injury after cardiac arrest between adult and pediatric PCAS patients. Because a few basic studies suggest the possibility that tolerance to ischemia-reperfusion brain injury may be dependent on age [37] and that there might be some potential molecular mechanisms specifically in pediatric PCAS patients that mediate the beneficial effects of TTM [38], it may not be surprising that the differences in treatment responsiveness to TTM according to the severity of PCAS may also differ between adult and pediatric PCAS patients. It would be of great interest to investigate these differences in the future.

There were some limitations in this study. First, our study was an observational study with a limited sample size. Although we used a nationwide prospective registry database of OHCA patients in Japan, the incidence of pediatric CA was quite low (2%) in the whole population. Second, we could not evaluate the effect of the setting core temperature during TTM because of the small sample size of the patients that received normothermia (only 26 patients with rCAST scores of ≤18.0 received normothermia treatment). Third, in this study, the endpoint used was the neurological outcome at 30 days. Although the outcome at 30 days has also been used in many other studies [39,40], we believe that it may be better to set a longer-term endpoint, e.g., the neurological outcome at 90 days, for more precise evaluation of the outcomes of PCAS [41]. Finally, the existence of a selection bias cannot be completely excluded because the choice of whether or not to perform TTM was left to the discretion of the treating hospital, even though we believe that all of the participating hospitals complied with the recommendation of the Japanese resuscitation guidelines in regard to the indications for TTM [22]. It would be of great interest to conduct a randomized, controlled trial to prove the efficacy of TTM in pediatric PCAS patients with rCAST scores of ≤18.0.

## 5. Conclusions

TTM was significantly associated with good neurological outcomes at 30 days in pediatric PCAS patients with rCAST scores of ≤18.0. All patients with rCAST scores of ≥18.5 showed poor neurological outcomes at 30 days, regardless of whether they received TTM or not.

## Figures and Tables

**Table 1:** Baseline characteristics of the patients.

Variables	TTM Group (N = 91)	No-TTM Group (N = 216)	*p*-Value
Age, yr	13.0 (2.0–16.0)	9.0 (1.0–15.0)	0.87
Sex, male, n (%)	65 (71.4)	138 (63.9)	0.20
Primary cause of OHCA, n (%)			0.01
Cardiovascular	36 (39.6)	50 (23.1)	
Respiratory	5 (5.5)	11 (5.1)	
Exogenous	42 (46.2)	115 (53.2)	
Other/Unknown	8 (8.8)	40 (18.5)	
Bystander and witnessed, n (%)	54 (59.3)	106 (49.1)	0.10
Chest compression by bystander, n (%)	56 (61.5)	132 (61.1)	0.94
AED by bystander, n (%)	10 (11.0)	14 (6.5)	0.19
Initial rhythm, n (%)			<0.001
VF or VT	24 (26.4)	12 (5.6)	
PEA	19 (20.9)	65 (30.1)	
Asystole	35 (38.5)	111 (51.4)	
Unknown	13 (14.3)	28 (13.0)	
Time until ROSC, min	34.0 (18.0–47.0)	40.0 (27.0–51.0)	0.008
GCS_M ≥ 2, n (%)	9 (9.9)	24 (11.1)	0.75
Use of ECMO, n (%)	16 (17.6)	11 (5.1)	<0.001
rCAST score, points	16.0 (11.5–18.0)	16.5 (15.0–18.5)	0.12
Good neurological outcome at 30 days, n (%)	26 (28.6)	23 (10.7)	<0.001

Data are presented as the median and interquartile ranges (25–75% percentile) or as absolute frequencies with percentages. CA = cardiac arrest; AED = automated external defibrillator; VF = ventricular fibrillation; VT = ventricular tachycardia; PEA = pulseless electrical activity; ROSC = return of spontaneous circulation; GCS = Glasgow coma scale; ECMO = extracorporeal membrane oxygenation; rCAST = revised post-cardiac arrest syndrome for therapeutic hypothermia.

## Data Availability

The datasets used and analyzed during the current study are available from the corresponding author on reasonable request.

## Supplementary Materials

The following supporting information can be downloaded at https://www.mdpi.com/article/10.3390/life15010026/s1: Figure S1: Receiver operating characteristic curve of the rCAST score. The dark red line indicates the ROC curve of rCAST for good neurological outcome at 30 days. ROC = receiver operating characteristic; rCAST = revised post-cardiac arrest syndrome for therapeutic hypothermia; AUC = area under the ROC cure; CI = confidence interval; Figure S2: Calibration plots of the rCAST score and predicted probability by each score point on rCAST score. Calibration plots of the rCAST score for neurological outcome at 30 days. The calibration curves represent the relationship between the mortality predicted by the rCAST score (*x*-axis) and the observed outcome (*y*-axis). The gray lines in figures represent perfect calibration. rCAST = revised post-cardiac arrest syndrome for therapeutic hypothermia.
