# Supplementary material for: Effect Size of Targeted Temperature Management in Pediatric Patients with Post-Cardiac Arrest Syndrome According to the Severity
Source: Life (Basel). 2024 Dec 30;15(1):26. doi: 10.3390/life15010026 (PMC11767084; doi:10.3390/life15010026)
Supplement: Supplementary file 1 [file life-15-00026-s001.zip › Supplemental Table2.pdf]

Supplementary Table S2. Baseline Characteristics of the Patients not received TTM by rCAST quintile

Variables	First quintile (N = 36)	Second quintile (N = 31)	Third quintile (N = 55)	Fourth quintile (N = 21)	Fifth quintile (N = 73)
Age, yr	13.0 (8.3-15.0)	13.0 (5.0-16.0)	6.0 (1.0-14.0)	10.0 (3.5-13.5)	4.0 (0-15.0)
Sex, male, n (%)	26 (72.2)	18 (58.1)	38 (69.1)	10 (47.6)	46 (63.0)
Primary cause of CA, n (%)					
Cardiovascular	20 (55.6)	8 (25.8)	7 (12.7)	5 (23.8)	10 (13.7)
Respiratory	1 (2.8)	1 (3.2)	4 (7.3)	2 (9.5)	3 (4.1)
Exogenous	12 (33.3)	17 (54.8)	32 (58.2)	13 (61.9)	41 (56.2)
Other/Unknown	3 (8.3)	5 (16.1)	12 (21.8)	1 (4.8)	19 (26.0)
Bystander & witnessed, n (%)	32 (88.9)	24 (77.4)	50 (90.9)	0 (0)	0 (0)
Chest compression by bystander, n (%)	26 (72.2)	12 (38.7)	30 (54.5)	14 (66.7)	50 (68.5)
AED by bystander, n (%)	12 (33.3)	1 (3.2)	0 (0)	1 (4.8)	0 (0)
Initial rhythm, n (%)					
VF or VT	6 (16.7)	6 (19.4)	0 (0)	0 (0)	0 (0)
PEA	10 (27.8)	13 (41.9)	18 (32.7)	3 (14.3)	21 (28.8)
Asystole	0 (0)	10 (32.3)	31 (56.4)	18 (85.7)	52 (71.2)
Unknown	20 (55.6)	2 (6.5)	6 (10.9)	0 (0)	0 (0)
Time until ROSC, min	11.5 (2.5-22.8)	38.0 (26.0-46.0)	45.0 (35.0-56.0)	39.0 (24.5-53.0)	44.0 (36.0-54.0)
GCS_M ≥2, n (%)	23 (63.9)	1 (3.2)	0 (0)	0 (0)	0 (0)
Use of ECMO, n (%)	1 (2.8)	4 (12.9)	4 (7.3)	0 (0)	2 (2.7)

Data are presented as the median and interquartile ranges (25-75% percentile) or as absolute frequencies with percentages.

CA = cardiac arrest, AED = automated external defibrillator, VF = ventricular fibrillation, VT = ventricular tachycardia, PEA = pulseless electrical activity, ROSC = return of spontaneous circulation, GCS = Glasgow coma scale, ECMO = extracorporeal membrane oxygenation.
