# Supplementary material for: Effect Size of Targeted Temperature Management in Pediatric Patients with Post-Cardiac Arrest Syndrome According to the Severity
Source: Life (Basel). 2024 Dec 30;15(1):26. doi: 10.3390/life15010026 (PMC11767084; doi:10.3390/life15010026)
Supplement: Supplementary file 1 [file life-15-00026-s001.zip › Supplementary Table1.pdf]

Supplementary Table S1. Baseline Characteristics of the Patients received TTM by rCAST quintile

Variables	First quintile (N = 26)	Second quintile (N = 19)	Third quintile (N = 17)	Fourth quintile (N = 9)	Fifth quintile (N = 20)
Age, yr	16.5 (12.0-17.0)	14.0 (5.0-15.0)	5.0 (1.0-14.0)	14.0 (9.0-16.5)	1.0 (0-10.0)
Sex, male, n (%)	22 (84.6)	13 (68.4)	11 (64.7)	7 (77.8)	12 (60.0)
Primary cause of CA, n (%)					
Cardiovascular	20 (76.9)	8 (42.1)	2 (11.8)	3 (33.3)	3 (15.0)
Respiratory	0 (0)	2 (10.5)	2 (11.8)	0 (0)	1 (5.0)
Exogenous	6 (23.1)	8 (42.1)	9 (52.9)	5 (55.6)	14 (70.0)
Other/Unknown	0 (0)	1 (5.3)	4 (23.5)	1 (11.1)	2 (10.0)
Bystander & witnessed, n (%)	22 (84.6)	16 (84.2)	16 (94.1)	0 (0)	0 (0)
Chest compression by bystander, n (%)	20 (76.9)	6 (31.8)	8 (47.0)	7 (77.8)	15 (75.0)
AED by bystander, n (%)	8 (30.8)	0 (0)	0 (0)	2 (22.2)	0 (0)
Initial rhythm, n (%)					
VF or VT	11 (42.3)	9 (47.4)	0 (0)	4 (44.4)	0 (0)
PEA	5 (19.2)	1 (5.3)	6 (35.3)	3 (33.3)	4 (20.0)
Asystole	2 (7.7)	6 (31.6)	9 (52.9)	2 (22.2)	16 (80.0)
Unknown	8 (30.8)	3 (15.8)	2 (11.8)	0 (0)	0 (0)
Time until ROSC, min	16.0 (9.0-31.5)	36.0 (26.0-60.0)	39.0 (29.5-53.5)	52.0 (31.5-65.5)	37.0 (31.0-46.8)
GCS_M ≥2, n (%)	8 (30.8)	1 (5.3)	0 (0)	0 (0)	0 (0)
Use of ECMO, n (%)	2 (7.7)	7 (36.8)	4 (23.5)	3 (33.3)	0 (0)
Therapeutic hypothermia, n (%)	16 (61.5)	15 (78.9)	9 (52.9)	5 (55.6)	13 (65.0)
Setting core temperature during TTM*					
32 °C	0 (0)	0 (0)	0 (0)	1 (11.1)	0 (0)
33 °C	0 (0)	1 (5.3)	0 (0)	0 (0)	1 (5.0)
34 °C	16 (61.5)	14 (73.7)	9 (52.9)	4 (44.4)	12 (60.0)
35 °C	2 (7.7)	1 (5.3)	2 (11.8)	1 (11.1)	4 (20.0)

36 °C	8 (30.8)	3 (15.8)	6 (35.3)	2 (22.2)	3 (15.0)
Cooling method for TTM					
gastric cooling	2 (7.7)	0 (0)	0 (0)	0 (0)	1 (5.0)
cold intravenous fluid	8 (30.8)	5 (26.3)	4 (23.5)	0 (0)	4 (20.0)
surface cooling with feedback system	20 (76.9)	11 (57.9)	10 (58.8)	6 (66.7)	16 (80.0)
intravascular cooling with feedback system	4 (15.4)	9 (47.4)	6 (35.3)	1 (11.1)	0 (0)
others/unknown	1 (3.8)	1 (5.3)	2 (11.8)	3 (33.3)	0 (0)

Data are presented as the median and interquartile ranges (25-75% percentile) or as absolute frequencies with percentages.

CA = cardiac arrest, AED = automated external defibrillator, VF = ventricular fibrillation, VT = ventricular tachycardia, PEA = pulseless electrical activity, ROSC = return of spontaneous circulation, GCS = Glasgow coma scale, ECMO = extracorporeal membrane oxygenation, TTM = targeted temperature management.

*A case in the group of fourth quintile missed value for a setting core temperature.
