# Supplementary figures and images for: Effect Size of Targeted Temperature Management in Pediatric Patients with Post-Cardiac Arrest Syndrome According to the Severity
Source: Life (Basel). 2024 Dec 30;15(1):26. doi: 10.3390/life15010026 (PMC11767084; doi:10.3390/life15010026)

Supplemental Figure 1. Receiver operating characteristic curve of rCAST score.

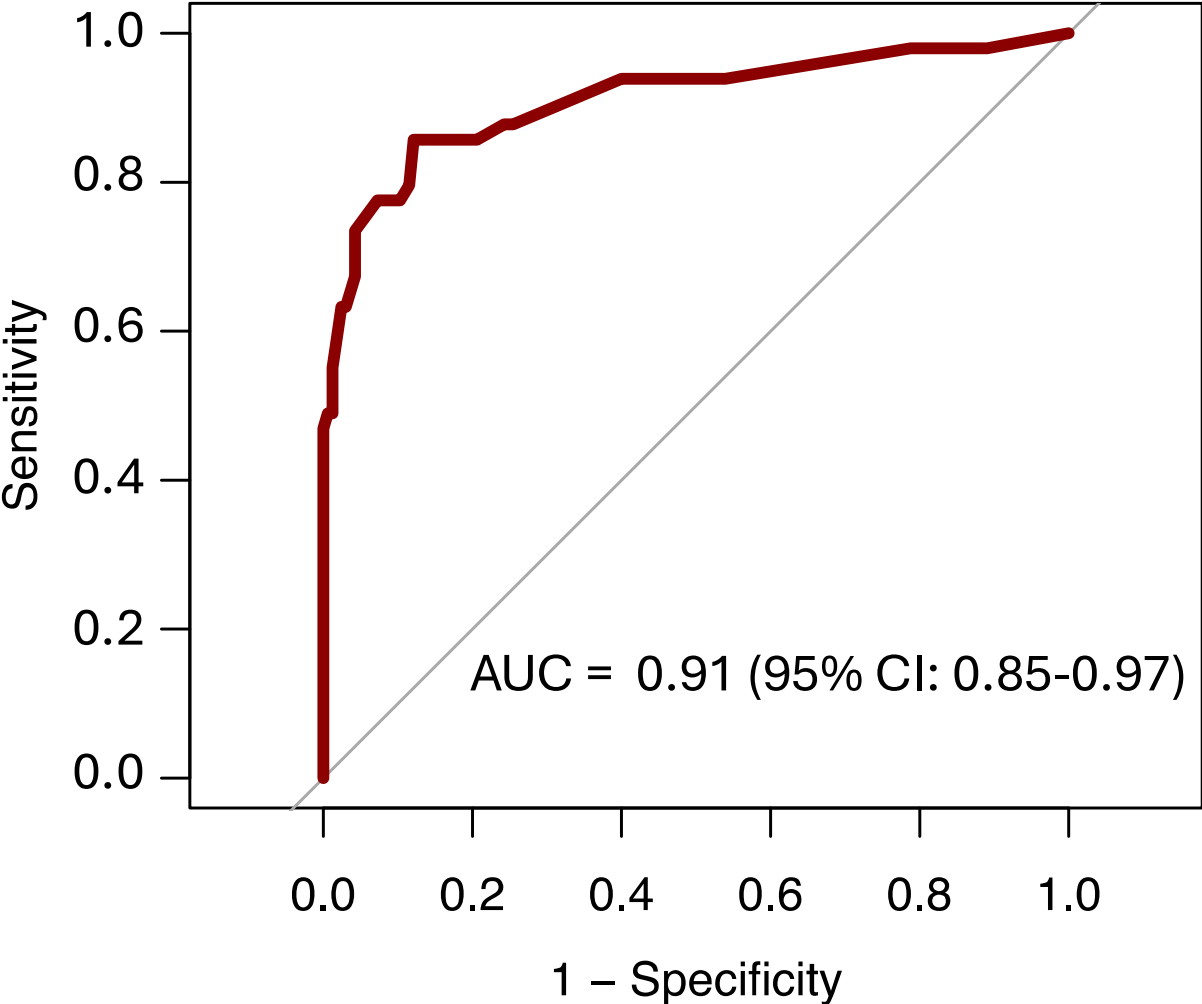

Supplement: Supplementary file 1 [file life-15-00026-s001.zip › Supplement Figure1.pdf]
